# Usability of the international HAVNet hepatitis A virus database for geographical annotation, backtracing and outbreak detection

**DOI:** 10.2807/1560-7917.ES.2018.23.37.1700802

**Published:** 2018-09-13

**Authors:** Annelies Kroneman, Rita de Sousa, Linda Verhoef, Marion P G Koopmans, Harry Vennema, on behalf of the HAVNet network

**Affiliations:** 1 National Institute for Public Health and the Environment (RIVM), Bilthoven, the Netherlands; 2 National Institute of Health Dr. Ricardo Jorge, Lisbon, Portugal; 3 Office for Risk Assessment and Research, Netherlands Food and Consumer Product Safety Authority (NVWA), Utrecht, the Netherlands (current affiliation); 4 Erasmus Medical Centre, Rotterdam, the Netherlands; 5 The members of the HAVNet network are acknowledged at the end of the article

**Keywords:** hepatitis A virus, molecular typing, database, genotype, cluster analysis

## Abstract

HAVNet is an international laboratory network sharing sequences and corresponding metadata on hepatitis A virus in an online database. **Aim:** We give an overview of the epidemiological and genetic data and assess the usability of the present dataset for geographical annotation, backtracing and outbreak detection. **Methods:** A descriptive analysis was performed on the timeliness, completeness, epidemiological data and geographic coverage of the dataset. Length and genomic region of the sequences were reviewed as well as the numerical and geographical distribution of the genotypes. The geographical signal in the sequences was assessed based on a short common nt stretch using a 100% identity analysis. **Results:** The 9,211 reports were heterogeneous for completeness and timeliness, and for length and genomic region of the sequences. Some parts of the world were not represented by the sequences. Geographical differences in prevalence of HAV genotypes described previously could be confirmed with this dataset and for a third (1,075/3,124) of the included sequences, 100% identity of the short common sequence coincided with an identical country of origin. **Conclusion:** Analysis of a subset of short, shared sequences indicates that a geographical annotation on the level of individual countries is possible with the HAVNet data. If the current incompleteness and heterogeneity of the data can be improved on, HAVNet could become very useful as a worldwide reference set for geographical annotation and for backtracing and outbreak detection.

## Introduction

Hepatitis A virus (HAV) belongs to the genus *Hepatovirus* within the *Picornaviridae* family. There is only one recognised serotype of HAV. Based on partial genomic sequences, three human genotypes (I to III) have been identified, with distinct geographical distribution [1,2].

The virus causes acute liver disease and is transmitted through ingestion of contaminated food and water or through direct contact with a contagious person. Both infection and vaccination lead to lifelong immunity. Incidence is strongly correlated with socioeconomic indicators and access to safe drinking water, and has substantially decreased in recent decades with improvements in sanitation and hygienic conditions, combined, in some regions, with childhood vaccination [3,4]. The level of endemicity of HAV was estimated for the 21 Global Burden of Disease (GBD) world regions, with four levels distinguished: (i) high, ≥ 90% of the population have immunity by the age of 10 years; (ii) intermediate, ≥ 50% have immunity by the age of 15 years; (iii) low, ≥ 50% have immunity by the age of 30 years; (iv) very low, < 50% have immunity by the age of 30 years [5-7]. In high-endemicity areas, nearly all children become infected at a very young age, and most adults are protected.

High endemicity is still present in some developing countries, mostly in Africa, and in the Middle East, Asia and Central and South America [5,6].

In very low-endemicity countries, groups at increased risk for hepatitis A are mainly international non-vaccinated travellers, men who have sex with men (MSM), and people who use drugs intravenously [8]. Apart from these risk groups, there is a general risk of infection through the consumption of contaminated, imported foods. Due to the globalisation of the food market, this risk is increasing [9-11]. Countries producing and exporting fruit, vegetables and shellfish products may have a higher level of HAV endemicity than the countries importing the products. As the virus is very stable in the environment, and the dose required to infect susceptible humans is low, an outbreak is likely to occur when contaminated food is introduced into a large, susceptible population [12]. Added to this risk is the fact that the virus is excellently preserved in frozen, ready-to-eat products [13], which may be shipped globally.

Due to the long incubation period for hepatitis A (15–50 days), linking and traceback of food-borne clusters or outbreaks of HAV are not straightforward. Given the stability of HAV, molecular typing and comparison of HAV strains can help to identify outbreaks and link cases, particularly in outbreaks with foods that have been dispersed over a wide geographic region. This requires international sharing of combined epidemiological and laboratory data [14].

Over 7,000 HAV sequences (including simian sequences) are available in the open-sequence repository of GenBank [15], but structured registration of epidemiological data such as source or country of infection is lacking. In 2010, the international hepatitis A virus laboratory network, HAVNet, was set up and a database of HAV sequences combined with epidemiological data was implemented, starting from the database used in the former European project, the Food-borne viruses in Europe Network [16-18]. Here, we describe the data currently available in the HAVNet database. The completeness of the epidemiological data is evaluated and the available sequences are analysed for length, genomic region and genetic diversity. These results are used to assess the usefulness of the present dataset for geographical annotation, backtracing and outbreak detection.

## Methods

### HAVNet network, database and typing tool

The HAVNet network consists of virologists from universities and public health institutes [19] using an online password-protected database platform. The database is used on a give-and-take basis which involves signing a confidentiality agreement. Besides the sequences, a set of background data fields is available (e.g. sampling date, age, sex, HAV vaccination status, day of onset of disease, suspected country of infection, suspected transmission route and implicated food). As not all laboratories are able to submit epidemiological data, the only compulsory fields are case identifier and year of sampling. The platform offers online analysis and visualisation tools such as the Basic Local Alignment Search Tool (BLAST), phylogeny and a geographical analysis tool. HAV sequences available in the public domain (GenBank [15]) are added to the dataset on a regular basis. If not available as standard in the GenBank record, the sampling date and geographic origin of the sequences are supplemented after searching the GenBank record and associated publications.

Standardised genotyping of all sequences in the database is realised through linkage with the newly developed HAV genotyping tool [20] through a web service. This tool was developed using building blocks from the open source REGA HIV-1 subtyping tool [21], analogous to the norovirus and enterovirus typing tools [22]. The typing algorithm starts with a BLAST analysis of the query sequence against a reference set of sequences from viruses in the family *Picornaviridae*, resulting in identification as HAV, and assigning coordinates of the genomic region of the query sequence, relative to the public reference sequence (NC_001489) [23]. The second step is a phylogenetic analysis of the query sequence compared with a subset of HAV reference sequences, to assign the genotype, with profile alignment, construction of phylogenetic trees and bootstrap validation. The genotyping result is subsequently stored in the HAVNet database. A download of the complete HAVNet dataset on 27 February 2017 was used for the analyses, including the additional and manually supplemented GenBank entries.

### Descriptive analysis of the HAVNet dataset

A download of the complete HAVNet dataset on 27 February 2017 was used for the analyses, containing 9,211 reports, with 9,783 sequences (572 reports reported two sequences, from two different genomic regions), of which 2,426 reports were submitted by 18 HAVNet members and 6,785 were retrieved from GenBank (Table 1). The earliest reported year of sampling for the GenBank sequences was 1957, and for the HAVNet sequences it was 2000.

**Table 1:** Hepatitis A virus sequences reported to HAVNet by participating countries and reported to GenBank, based on year of sampling, pre-2010−2017 (n = 9,211)

Source of reports	Sampling year										Total
	Unknown	< 2010	2010	2011	2012	2013	2014	2015	2016	2017	
GenBank	2,051	3,305	395	113	265	470	147	38	1	0	6,785
Australia	0	0	16	13	23	30	36	23	0	0	141
Austria	0	0	0	0	0	0	2	0	0	0	2
Canada	0	0	0	0	0	0	0	3	1	0	4
Czech Republic	0	56	0	0	0	0	0	0	0	0	56
Finland	0	0	0	0	0	0	0	5	0	0	5
France	0	51	0	0	0	0	0	1	0	0	52
Germany	0	2	0	6	13	17	34	11	47	32	162
Hungary	0	178	7	2	1	4	0	0	0	0	192
Ireland	0	0	0	0	1	32	5	15	2	0	55
Israel	0	0	0	0	0	2	0	0	0	0	2
Italy	0	0	0	0	0	0	4	3	0	0	7
Latvia	0	100	0	0	0	0	0	0	0	0	100
The Netherlands	0	697	212	98	82	78	88	56	63	19	1,393
New Zealand	0	0	0	0	0	0	0	4	0	0	4
Norway	0	0	0	0	2	26	0	0	0	0	28
Spain	0	49	0	0	0	0	0	0	0	0	49
Sweden	0	43	0	0	0	3	11	29	15	3	104
United Kingdom	0	0	0	4	2	3	3	58	0	0	70
** Total**	**2,051**	**6,532**	**630**	**236**	**389**	**665**	**330**	**246**	**129**	**54**	**9,211**

HAVNet: hepatitis A virus Network.

The completeness of the dataset was scored for information on sampling date, suspected geographical origin of the virus, possible source of infection, vaccination status and hospitalisation. For the subset of HAVNet reports for which a complete sampling date was available, and which were submitted after 2010 (the year in which the HAVNet database was started and historical datasets were uploaded) the reporting lag was calculated. Geographic distribution of genotypes was compared with previously published data [1,2]

### Analysis of 100 nt sequence types for geographical signal

For the analysis of the geographical signal, they were evaluated for their lengths and coordinates relative to the reference sequence. Based on these findings, a target region for the analysis was chosen encompassing the majority of the sequences in the database, while remaining of sufficient length to have suitable discriminatory power in the subsequent analysis.

The subset of sequences included in this analysis comprises all entries of human HAV in the HAVNet database reporting the most likely geographical origin of the infection. Only sequences from endemic countries were included.

The large heterogeneity of the sequence regions in the dataset resulted in the choice of a short target region of only 100 overlapping nt. With such short sequences, a reliable phylogenetic cluster analysis is not possible. Thus, a very basic classification method was chosen in which sequences were grouped on the basis of 100% identity within this 100 nt region, i.e. identical sequence-type. Minimum group size was arbitrarily set at three. Groups of two were left out because despite thorough curation, duplicate sequences in the HAVNet dataset cannot be excluded due to the two sources (HAVNet and GenBank) of the sequences.

**Figure 1:**
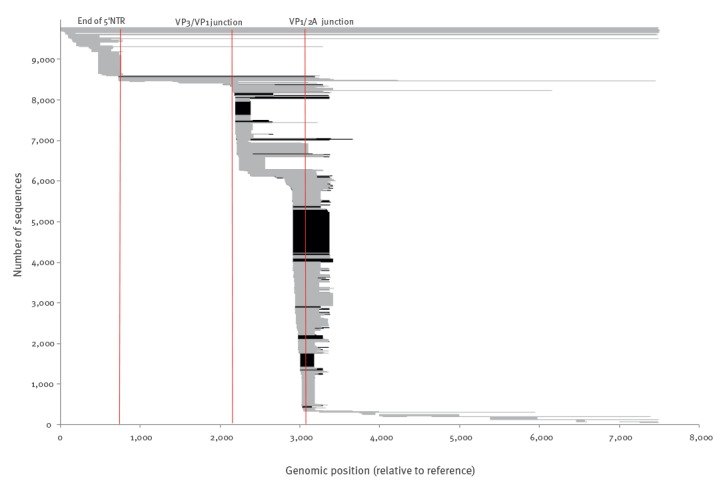
Representation of the genomic coordinates of hepatitis A virus sequences in the HAVNet database relative to the reference, 1957−2017 (n = 9,783)

**Figure 2:**
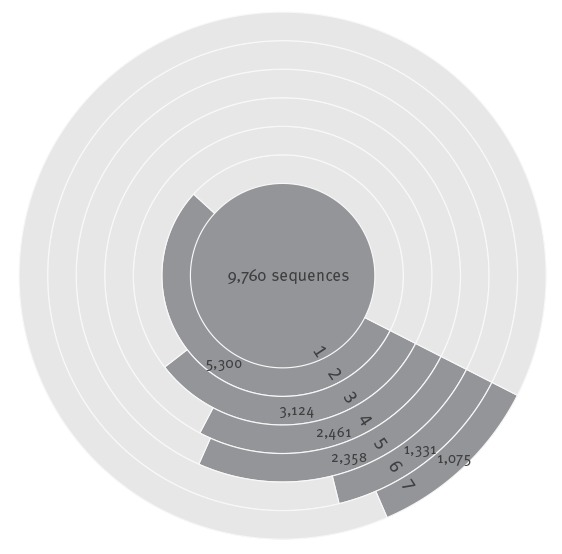
Specification of the use of the 9,760 human hepatitis A virus sequences in the analyses

**Figure 3:**
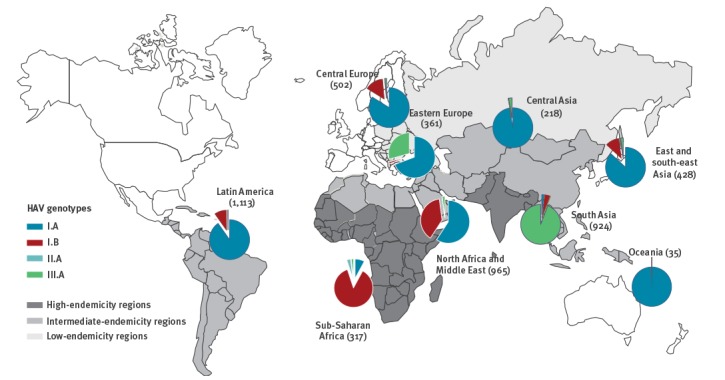
Distribution of human hepatitis A virus genotypes over the endemic Global Burden of Disease regions, pre-2010−2017 (n = 4,863)

Because of the overrepresentation of some countries in the database, for each sequence-type group the geographic information was analysed by comparing the frequencies of the suspected country of origin for sequences in that group with the geographic distribution of the entire dataset. The p value thus represents the probability of finding this frequency based on chance. P values below 0.05 were considered statistically significant. Calculations were performed and Figures 1, 2 and part of Figure 3 were composed using Microsoft Excel (Microsoft Corporation, Redmond, Washington, United States, 2010).

HAVNet: hepatitis A virus Network.

The x axis represents the position of each sequence relative to the reference HAV genome NC_001489. Each horizontal grey line is one sequence from GenBank, each black line a sequence reported by a HAVNet member. The vertical red lines demarcate major typing regions: the end of the 5’ non-coding region (position 735), the VP3/VP1 junction (between positions 2,207 and 2,208) and the VP1/2A junction (between position 3,107 and 3,108).

1: All sequences of human origin.

2: From 96 endemic countries.

3: Covering the specified 100nt region, including 92 endemic countries.

4: 100% identical to ≥ 2 others (= sequence type group).

5: Significantly associated to one of the 181 sequence type groups, including 74 countries.

6: In 145 groups from a single GBD region.

7: In 122 groups from a single country, including 29 countries.

HAVNet: hepatitis A virus Network.

This map is based on the suspected origin of the virus as reported by HAVNet members or determined from the GenBank record or associated publications.

The number of sequences per region are shown in parentheses. Reports from regions of very low endemicity have been omitted.

## Results

### Descriptive analysis of the HAVNet epidemiological data

The 2,426 reports submitted by HAVNet members were evaluated for epidemiological data. Of these, 972 (40%) were reported with a possible source of infection (Table 2). Of the reports with a reported source of infection, 508 (52%) reported travel to an endemic country, and 77 (8%) reported a possible food-borne source, of which 16 reported shellfish, 18 fresh or frozen fruit, and 43 other or unknown food. Fewer reports held other epidemiological data: only 282 (12%) reports from HAVNet members recorded the vaccination status, and 79 (3%) recorded whether or not the patient was hospitalised (data not shown).

**Table 2:** Reported source of hepatitis A in HAVNet reports, 2000−2017 (n = 972)

Possible source of infection	Number of reports
Contact with hepatitis A patient	241
Contact with traveller with hepatitis A	5
Homeless/PWID	13
MSM	115
Travel to endemic country	508
Food-borne: fresh or frozen soft fruit	18
Food-borne: shellfish	16
Food-borne: other/unknown	43
Waterborne	2
Other	11
**Total**	**972**

HAV: hepatitis A virus; HAVNet: hepatitis A virus Network; MSM: men who have sex with men; PWID: people who inject drugs.

For HAVNet reports with complete sampling dates and reported after 2010 (start of the present HAVNet database platform), the reporting lag was computed: of the 850 reports included, the median reporting lag was 94 days (min = 0 days, max = 1,282 days), with large differences per country and no clear trend over the years.

### Genetic data

The 9,783 sequences in the database ranged in length from 60 nt to around 7,500 nt, the length of the complete genome. The genomic region covered by the sequences is shown in Figure 1.

The two genomic regions most widely used for genotyping, the VP1/2A region and the VP3/VP1 region [1,24], were predominant in the dataset. A limited set of complete genomes was available. These were mainly downloaded from GenBank.

Of the 9,211 reports, 8,657 (2,394 reported by HAVNet members, 6,263 retrieved from GenBank) could be typed as human HAV genotypes by the typing tool. Twenty three were of genotype V, which is not found in humans, so these were excluded from the overview in Table 3, as were the sequences for 531 reports which could not be typed by the typing tool. Of these, 50% (267) were sequences from the 5’ non-translated region, while only 9% of the typed sequences were from that region. One was a hepatitis B virus sequence. In general, the sequences which could not be genotyped were shorter than the typed sequences, with a median length of 196 nt, vs 332 nt for the typed sequences. They tended to cluster clearly with one genotype (I, II or III), but could not reliably be assigned to one of the subtypes within each genotype and thus remain unassigned due to the present setup of the typing tool.

**Table 3:** Human hepatitis A virus genotypes in the dataset of Robertson et al. compared with the HAVNet and GenBank datasets

HAV genotype	Dataset					
	Robertson et al. [1]		GenBank		HAVNet	
	**n**	**Ratio (%)**	**n**	**Ratio (%)**	**n**	**Ratio (%)**
I.A	71	68.9	3,924	62.7	1,339	55.9
I.B	12	11.7	1,084	17.3	780	32.6
II	1	1.0	30	0.5	4	0.2
III.A	13	12.6	1,201	19.2	271	11.3
III.B	6	5.8	24	0.4	0	0.0
**Total**	**103**	**100**	**6,263**	**100**	**2,394**	**100**

HAV: hepatitis A virus; HAVNet: hepatitis A virus Network.

The majority (7,127, 82%) of the 8,657 reports in the download were genotype I (88.5% for HAVNet, 80.0% for GenBank) (Table 3). In the HAVNet dataset, the proportion of I.A was smaller than in the GenBank dataset: 55.9% vs 62.7%, and for I.B vice versa: 32.6% vs 17.3%. The next most common genotype was III.A, with 19.2% in the GenBank sequences, and 11.3% for HAVNet. Genotype II was very rare, with only 34 reports in total. All 24 III.B sequences in the download dataset were retrieved from GenBank and had sampling dates before 1990 (data not shown).

### Geographical distribution of hepatitis A virus genotypes

For 5,300 of the 9,760 sequences (54.3%) in the dataset, the endemic country from which the virus originated was present (Figure 2). Figure 3 shows the distribution of the human HAV genotypes over the GBD world regions with low to high endemicity [2]. The geographical distribution of genotypes is in concordance with the findings in 1992 by Robertson et al. [1]. Genotype I.B is predominant in sub-Saharan Africa, I.A in South and Central America and III.A in south Asia. In North Africa and the Middle East both I.A and I.B can be found. Based on five sequences, Robertson et al. [1] only found I.A in this region.

### Geographical signal in the sequences

The most predominant genomic region in the database was a region of 100 nt at the VP1/2A region (positions 3,046–3,146 compared with NC_001489) (Figure 1).

A multiple alignment was made with all 3,187 sequences covering this genomic region, for which an endemic country was reported as the possible origin of the virus. A set of 63 sequences was excluded due to unreliable alignment in the selected genomic region.

This resulted in a subset of 3,124 sequences (32% of the downloaded dataset) from 92 of 96 endemic countries present in the database (95%), for the analysis of geographical signal (Figure 2).

On the basis of 100% identity within this 100 nt region, 2,461 sequences (79% of the subset of sequences included in the analysis) were assigned to 181 sequence-type groups of three or more sequences, 2,358 of which were significantly associated with this group.

Of these, 122 (67%) sequence-type groups contained sequences from a single country. Of the 59 sequence types from more than one country, 23 came from countries within the same GBD world region. Seventy four countries (75% of all endemic countries represented in the database) were significantly associated with at least one of the sequence types. The 22 countries without significant association to a sequence type were all present in the dataset with only one or two sequences (data not shown).

Of the 663 sequences (21%) which were not part of a sequence-type group with more than two members, 477 sequence types were unique, and 186 had two representatives.

There were 118 sequence-type groups of genotype I.A, 46 of I.B, 1 of II.B and 17 of III.A (Table 4). The III.A groups were more often composed of more than one country, despite their smaller average size.

**Table 4:** Hepatitis A virus genotype distribution and composition of the sequence-type groups, pre-2010−2017 (n = 2,461 sequences)

HAV genotype	Average group size	Number of groups	Single country groups	Multiple country groups
**I.A**	**15.1**	**118**	**83**	**35**
I.B	11. 6	46	31	15
II.A	4.0	1	0	1
III.A	8.5	17	8	9

HAV: hepatitis A virus.

## Discussion

Analysis of the current global dataset of sequence data for HAV shows that the HAVNet database is promising for source tracking; however, improvements in data quality are necessary to cover gaps regarding essential metadata and to enable optimal and timely use of such data in outbreak studies. In many reports, essential metadata are missing. In addition, parts of the world are well-covered with respect to reports and sequences, while other parts of the world are missing. When available, comparison of sequences is complicated by different genomic regions targeted for the sequences that are shared.

With the current lag times for reporting, the database is able to serve as an outbreak detection tool for frozen foods with long shelf lives. For fresh produce, however, timeliness requires improvement, as, combined with the long incubation period for HAV, it is outside the actionable range.

Nevertheless, despite its present sub-optimal form, the HAVNet database has illustrated its use for overcoming public health issues in HAV source tracing. The data collected within the network supported the detection and source-tracing of several international outbreaks, food-borne as well as a recent large outbreak among MSM [11,14,25-28].

Using the current HAVNet dataset, both the ratio of the different genotypes and the worldwide distribution of the different genotypes are largely very similar to the findings of the 1992 inventory [1]. Only genotype III.B is relatively underrepresented in the current dataset compared with the historic overview, and all III.B sequences in the database were several decades old. This may reflect a true decrease in global presence, as this genotype was strongly associated with Japan, which has seen a rapid decline in HAV prevalence [29]. As this country now has the lowest endemicity level, this genotype seems to have disappeared [5].

The lack of standardisation of partial genome sequencing precluded integral analysis of this data. Concessions had to be made with regard to the length of the included genomic region, which resulted in using only a fraction of the genetic information in the sequences. Still, our analyses did find a distinctive geographic signal in the 3,124 included sequences at the level of individual countries or GBD regions. In order to be able to validate this with a larger genomic region, the next step should be an international harmonisation of sequencing protocols, thereby building a well-annotated set of reference sequences of sufficient length. Here, the use of complete genomes, as has been advocated for tracking of other pathogens [30-35], would be optimal, but due to the higher sequencing costs it is not yet realistic for all laboratories. Such a reference set could be used to build a second analysis step in the HAV typing tool after the genotyping, where geographical annotation could be performed, as an indication of the country or region of origin of the strain. Such geotagging would help to focus source tracing of a HAV infection, particularly in food-borne outbreaks where imported products are involved.

The shift in III.B prevalence shows the importance of regular and systematic updating of the reference sequence database. Moreover, shifts in endemicity and the ever-increasing levels of international travel and food trade might challenge the present geographical distribution of HAV strains and thus the use of the database for source tracing in the future.

Another important element is the need for global coverage: there are still blank spots on the world map, outlined on the basis of the HAVNet dataset, partly due to the absence of sequences from these regions, but also due to absence of sufficient background data to pinpoint the geographical origin of the sequences submitted. However, a growing number of laboratories are contributing sequences to the database, and we hope that in the near future these blank areas will be filled in, leading to a more complete representation of the sequence space of HAV.

Despite its shortcomings, the HAVNet database has proved promising in pinpointing the geographical origin of sources in outbreak investigations.

If the HAVNet database can be further improved, it could make use of other sources, or could be used at earlier stages of international outbreaks, such as the outbreak among MSM in Europe [26]. To optimise public health profit, the HAV community is encouraged to expand its genome sequencing efforts, in terms of genome coverage, metadata provision and timely sharing of information.
